# Anticitrullinated protein antibody (ACPA) in rheumatoid arthritis: influence of an interaction between *HLA-DRB1 shared epitope *and a deletion polymorphism in *glutathione s-transferase *in a cross-sectional study

**DOI:** 10.1186/ar3190

**Published:** 2010-11-18

**Authors:** Ted R Mikuls, Karen A Gould, Kimberly K Bynoté, Fang Yu, Tricia D LeVan, Geoffrey M Thiele, Kaleb D Michaud, James R O'Dell, Andreas M Reimold, Roderick Hooker, Liron Caplan, Dannette S Johnson, Gail Kerr, J Steuart Richards, Grant W Cannon, Lindsey A Criswell, Janelle A Noble, S Louis Bridges, Laura Hughes, Peter K Gregersen

**Affiliations:** 1 Omaha Veterans Affairs Medical Center and Nebraska Arthritis Outcomes Research Center, University of Nebraska Medical Center (UNMC), 986270 Nebraska Medical Center, Omaha, NE 68198-6270, USA; 2 Department of Genetics Cell Biology & Anatomy, UNMC, 985805 Nebraska Medical Center, Omaha, NE 68198-5805, USA; 3 Department of Biostatistics, UNMC, 984375 Nebraska Medical Center, Omaha, NE 68198-4375, USA; 4 Department of Medicine and Epidemiology, UNMC, 985300 Nebraska Medical Center, Omaha, NE 68198-5300, USA; 5 Department of Medicine, Dallas Veterans Affairs Medical Center, 4500 South Lancaster Road, Dallas, TX 75216-7191, USA; 6 Research, Denver Veterans Affairs Medical Center and the University of Colorado Denver, PO Box 6511, MS B115, Aurora, CO 80045, USA; 7 Department of Medicine, Jackson Veterans Affairs Medical Center and the University of Mississippi, 2500 North State Street, Jackson, MS 39216, USA; 8 Department of Medicine, Washington, DC, Veterans Affairs Medical Center and Georgetown University, Room 3A 161, 50 Irving Street NW, Washington, DC 20422, USA; 9 Department of Medicine, Salt Lake City Veterans Affairs Medical Center and the University of Utah, 50 North Medical Drive, Salt Lake City, UT 84132, USA; 10 Department of Medicine, University of California at San Francisco, Box 0500, 374 Parnassus Avenue 1st Floor, San Francisco, CA 94143-0500, USA; 11 Children's Hospital Oakland Research Institute, 5700 Martin Luther King Jr Way, Oakland, CA 94609, USA; 12 Department of Medicine, University of Alabama at Birmingham, 1530 3rd Avenue South, 178 SHEL, Birmingham, AL 35294-2182, USA; 13 Genomics and Human Genetics, Feinstein Institute Medical Research, 350 Community Drive, Manhasset, NY 11030, USA

## Abstract

**Introduction:**

A deletion polymorphism in *glutathione S-transferase Mu-1 (GSTM1-null) *has previously been implicated to play a role in rheumatoid arthritis (RA) risk and progression, although no prior investigations have examined its associations with anticitrullinated protein antibody (ACPA) positivity. The purpose of this study was to examine the associations of *GSTM1-null *with ACPA positivity in RA and to assess for evidence of interaction between *GSTM1 *and *HLA-DRB1 shared epitope (SE)*.

**Methods:**

Associations of *GSTM1-null *with ACPA positivity were examined separately in two RA cohorts, the Veterans Affairs Rheumatoid Arthritis (VARA) registry (*n *= 703) and the Study of New-Onset RA (SONORA; *n *= 610). Interactions were examined by calculating an attributable proportion (AP) due to interaction.

**Results:**

A majority of patients in the VARA registry (76%) and SONORA (69%) were positive for ACPA with a similar frequency of *GSTM1-null *(53% and 52%, respectively) and *HLA-DRB1 SE *positivity (76% and 71%, respectively). The parameter of patients who had ever smoked was more common in the VARA registry (80%) than in SONORA (65%). *GSTM1-null *was significantly associated with ACPA positivity in the VARA registry (odds ratio (OR), 1.45; 95% confidence interval (CI), 1.02 to 2.05), but not in SONORA (OR, 1.00; 95% CI, 0.71 to 1.42). There were significant additive interactions between *GSTM1 *and *HLA-DRB1 SE *in the VARA registry (AP, 0.49; 95% CI, 0.21 to 0.77; *P *< 0.001) in ACPA positivity, an interaction replicated in SONORA (AP, 0.38; 95% CI, 0.00 to 0.76; *P *= 0.050).

**Conclusions:**

This study is the first to show that the *GSTM1-null *genotype, a common genetic variant, exerts significant additive interaction with *HLA-DRB1 SE *on the risk of ACPA positivity in RA. Since GSTM1 has known antioxidant functions, these data suggest that oxidative stress may be important in the development of RA-specific autoimmunity in genetically susceptible individuals.

## Introduction

The human leukocyte antigen (*HLA*) region accounts for approximately one half of the genetic risk of rheumatoid arthritis (RA). This risk is attributable to alleles encoding a conserved amino acid sequence in the third hypervariable region of the *DRB1 *chain (commonly referred to as the *shared epitope *[*SE*]) [1]. Recent efforts have examined the importance of interactions of *SE *with other genetic and environmental factors in RA risk and progression. Most notably, studies have yielded evidence of significant interactions between *SE *and cigarette smoking in the development of anticitrullinated protein antibody (ACPA)-positive RA [2,3], although the precise mechanisms underpinning this interaction are not understood.

Genetic and environmental factors that mediate oxidative stress, including cigarette smoking, are postulated to play a central role in the pathogenesis of autoimmune disorders including RA. While oxidative stress represents a form of host defense, it can also result in tissue damage. Oxidative modification of proteins and other biologic molecules leads to the expression of neoantigens, a possible first step in the development of autoimmunity, which may herald the future onset of clinically relevant autoimmune disease [4]. Antioxidants, which mitigate tissue damage caused by reactive oxygen species, may serve important protective functions in RA. While not all studies have identified a similar protective effect [4,5], the dietary intake of small-molecule antioxidants has been reported to be inversely associated with RA risk [6-9]. Additionally, low circulating levels of antioxidants have been reported to portend the onset of RA [10].

In addition to the effects of exogenous antioxidants, oxidation is also regulated by several enzymes, including glutathione *S*-transferase (GST). A ubiquitous cytosolic protein, GST catalyzes the conjugation of glutathione to a variety of substrates, including reactive oxygen species and other toxins, facilitating their elimination. Four classes of GST have been identified: α, μ, π and θ. Approximately one half of all individuals of European ancestry are homozygous for a deletion at the *GST Mu-1 *(*GSTM1*) locus (*GSTM1-null*) [11] located on chromosome 1 (1p13.1).

The *GSTM1-null *genotype has been associated with an increased risk of RA and in most [12-14] but not all [15] case control studies. In addition to being implicated as a potential risk factor in RA, the *GSTM1-null *genotype is associated with higher levels of oxidative stress [16] and has been reported to be a risk factor for other smoking-related inflammatory diseases, including asthma, emphysema and atherosclerosis [17-21]. However, there have been no studies examining associations of *GSTM1 *genotypes with ACPA expression in patients with RA. This represents an important knowledge gap, since these antibodies are disease-specific, have significant prognostic and pathogenic significance and are increasingly recognized to characterize a unique subset of patients with RA [22,23]. In the present study, we have evaluated potential gene-gene interactions by exploring the *GSTM1*-null genotype as a risk factor for ACPA positivity in RA, providing evidence of an interaction with *HLA-DRB1 shared epitope (SE)*-containing alleles.

## Materials and methods

### Study subjects

All study subjects satisfied the American College of Rheumatology (ACR) criteria for RA [24] and were from two U.S. cohorts: the Veterans Affairs Rheumatoid Arthritis (VARA) registry [25] and the Study of New-Onset Rheumatoid Arthritis (SONORA) [26]. To limit population heterogeneity, analyses were limited to individuals self-reporting Caucasian race for whom banked samples and *HLA-DRB1 *data were available.

VARA is a multicenter registry with sites at nine VA medical centers in Brooklyn, NY; Dallas, TX; Denver, CO; Iowa City, IA; Jackson, MS; Omaha, NE; Portland, OR; Salt Lake City, UT; and Washington, DC. The registry has Institutional Review Board approval at each site, and patients provided informed written consent. Patients are eligible if they are U.S. Department of Veterans Affairs (VA) beneficiaries. SONORA includes patients with recent-onset RA enrolled within 12 months of diagnosis as part of a 5-year prospective follow-up study [26]. SONORA patients were recruited from 98 rheumatology practices in the U.S. and Canada, and all participants provided informed written consent. Variables abstracted from the corresponding data sets included age, gender and smoking status (never, former, or current). Smoking status in both cohorts was obtained using questionnaires reflecting exposure at the time of enrollment. Quantitative measures of smoking (pack-years and duration) were not routinely collected.

### Anticitrullinated protein antibody (ACPA)

Serum ACPA (immunoglobulin G (IgG)) was measured using second-generation enzyme-linked immunosorbent assays (ELISAs) in VARA (Diastat, Axis-Shield Diagnostics Ltd., Dundee, Scotland, UK; positive ≥5 U/ml) and SONORA (Inova Diagnostics, San Diego, CA, USA; positive ≥20 U/ml) using serum samples collected at enrollment.

### Determination of *GSTM1 *genotype

Primers "G2" and "G3" from a study by Brockmöller *et al*. [27] were used to amplify exons 3 through 5 of the *GSTM1 *gene using genomic DNA that was prepared from whole blood. These primers produce a 650-bp amplified fragment in individuals carrying at least one functional *GSTM1 *allele. This band is absent in *GSTM1-null *individuals because this mutation deletes exons 4 and 5. A 195-bp fragment of exon 7 of the *CYP1a1 *gene was used as an internal positive control for sample quality and polymerase chain reaction (PCR) using the primers described by Shields *et al*. [28]. Amplified products were resolved by electrophoresis through 1% agarose gels. Genotypes were scored independently by two investigators (KAG and KKB). On the basis of the empiric evidence for associations of this genotype across multiple conditions [17,29-31], individuals were categorized as *GSTM1-null *(homozygous for deletion) or *GSTM1-present *(one or two copies of functional allele). Individuals with an absent or faint *CYP1a1 *band (*n *= 8 from VARA and *n *= 25 from SONORA) were excluded from further analyses, leaving available data from 703 VARA individuals and 610 SONORA participants for analysis.

### Determination of *HLA-DRB1 *genotypes

In VARA, *HLA *genotyping was performed using one of two approaches: DNA sequencing of exon 2 using the AlleleSEQR HLA-DRB1 reagent kit and protocol (Abbott Molecular, Abbott Park, IL, USA) or with a PCR-based, sequence-specific oligonucleotide probe system. In the second of these methods, a series of oligonucleotide probes corresponding to known sequence motifs in *HLA*-*DRB1 *were immobilized onto a backed nylon membrane to create a "linear array." Exon 2 of *DRB1 *was amplified with a set of upstream biotinylated PCR primers corresponding to known sequence motifs in the first variable region of *DRB1 *and a single downstream biotinylated PCR primer that amplifies all alleles. This method specifically amplified only *DRB1 *genes and avoided amplification of other *DRB *genes. The PCR product was denatured and hybridized to the 81-probe *DRB1 *linear array. Arrays were incubated with streptavidin-horseradish peroxidase followed by tetramethylbenzidine. Images were created by placing the arrays on a flatbed scanner, and probe intensities were measured with proprietary software. Preliminary genotypes were determined, and data were then imported into Sequence Compilation and Rearrangement Evaluation software (SCORE(tm), QIAGEN, Valencia, CA, USA) for final genotyping and data export. The following were considered to be *DRB1 shared epitope *(*SE)*-containing alleles: *0101, *0102, *0104, *0105, *0401, *0404, *0405, *0408, *0409, *1001, *1402 and *1406.

In SONORA, all participants were *HLA-DRB1*-typed as previously described [32] initially using the sequence-specific oligonucleotide probes (SSOP) low-resolution method [33]. Individuals with DRB1 *04 and *01 were subsequently tested using a medium-resolution panel to allow for four-digit DRB1 subtyping.

### Statistical analyses

Associations of the *GSTM1-null *genotype with ACPA positivity were examined for each RA cohort using multivariate unconditional logistic regression. All analyses were adjusted for age (continuous variable) and gender to facilitate comparisons across the two divergent patient cohorts that differed based on these factors. Associations of *HLA-DRB1 SE *(positive vs. negative in addition to the number of *SE *alleles, 0 vs. 1 or 2) and smoking status modeled as ever versus never (and as current or former vs. never in a separate model) with ACPA positivity were also examined in separate analyses. Patients were then categorized on the basis of the presence of risk factor pairings (*GSTM1*-SE, *GSTM1*-smoking and smoking-*SE*), and associations of these risk factor assignments with outcomes were examined using similar regression techniques.

Gene-gene (*GSTM1-SE*) and gene-environment (*GSTM1-*smoking and *SE*-smoking) interactions were assessed with regard to ACPA positivity by examining for evidence of departure from additivity using the methods described by Rothman *et al*. [34]. Three-way interactions were not examined. Using this approach, an attributable proportion (AP) due to interaction (AP = 0 corresponds to no interaction, and AP = 1.0 corresponds to "complete" additive interaction) and 95% confidence intervals (CIs) were calculated, using the method of Hosmer and Lemeshow [35] to calculate the latter. The confidence interval serves as a statistical test of the interaction; if the null value (zero in this case) falls outside the interval, then the interaction is considered statistically significant. This method accounts for both the random variability and overlapping intervals in strata defined by the risk factors of interest [35]. Evidence of multiplicative interaction was examined by modeling the product term of interest. To optimize study power, assessments of interaction were limited to dichotomous variables (*SE-positive *vs. *SE-negative*, ever vs. never smoking) and to two-way interactions. All analyses were conducted using Stata version 10.0 software (Stata Corp., College Station, TX, USA).

## Results

### Patient characteristics

Patient characteristics are summarized in Table 1. Consistent with the demographic characteristics of VA beneficiaries nationally [36], VARA registry patients were predominantly men (93%) with a mean (± SD) age of 64 (± 11) years. In contrast, SONORA patients were younger, with a mean (SD) age of 53 (± 15) years, and were predominantly women (72%). A majority of patients were seropositive for ACPA (76% in VARA Registry and 69% in SONORA).

**Table 1:** Characteristics of rheumatoid arthritis study patients^a^

	Mean (SD) or number (%)	
	VARA (*n *= 703)	SONORA (*n *= 610)
Sociodemographics		
Age, yr^b^	64 (11)	53 (15)
Male gender^b^	655 (93%)	173 (28%)
		
ACPA-positive^b^	536 (76%)	420 (69%)
		
RA risk factors		
*HLA-DRB1 SE*-positive	531 (76%)	434 (71%)
One copy	356 (51%)	303 (50%)
Two copies	175 (25%)	131 (21%)
*GSTM1-null*	372 (53%)	315 (52%)
		
Smoking history^b^	(*n *= 693)	(*n *= 610)
Never	140 (20%)	213 (35%)
Former	371 (54%)	257 (42%)
Current	182 (26%)	141 (23%)

^a^ACPA, anticitrullinated protein antibody; *GSTM1, glutathione S-transferase Mu-1*; *SE, shared epitope*; SONORA, Study of New-Onset RA; VARA, Veterans Affairs Rheumatoid Arthritis Registry. ^b^*P *< 0.05 for differences between VARA and SONORA.

### Risk factor prevalence

The frequency of RA-related risk factors is shown in Table 1. The prevalence of at least one *HLA*-*DRB1 SE*-containing allele was similar in the VARA Registry (76%) and SONORA (71%) (*P *= NS). Approximately one half of patients (53% in the VARA Registry and 52% in SONORA) were *GSTM1-null *(*P *= NS), and a majority had a history of smoking, either current or former (80% in the VARA Registry and 65% in SONORA; *P *< 0.05).

### Age- and gender-adjusted associations

Associations of *GSTM1*, smoking, and *HLA-DRB1 *status with ACPA positivity in the VARA registry and SONORA are summarized in Table 2. In reference to patients with at least one functional *GSTM1 *allele, *GSTM1-null *was associated with a significantly higher odds ratio (OR) of ACPA positivity in the VARA Registry (OR, 1.45; 95% CI, 1.02 to 2.05), but not in SONORA (OR, 1.00; 95% CI, 0.71 to 1.42). There was a significant dose-related association of *HLA-DRB1 SE *with ACPA positivity in both cohorts, with more than 10-fold greater odds of ACPA positivity for those with 2 *SE *alleles compared with those with no *SE *allele (Table 2). In both cohorts, there were nonsignificant trends suggesting associations of current (vs. never) smoking with ACPA positivity, an effect that appeared to be more striking in the VARA registry (OR, 1.68; 95% CI, 0.98 to 2.88) than in SONORA (OR, 1.23; 95% CI, 0.76 to 1.99) (Table 2). Age- and gender-adjusted associations of composite risk factors with ACPA positivity are summarized in Table 3.

**Table 2:** Association of GSTM1-null, HLA-DRB1 shared epitope (SE) and smoking with ACPA positivity in rheumatoid arthritis^a^

	VARA (*n *= 703)			SONORA (*n *= 610)		
	ACPA+ (%)	OR (95% CI)	*P *value	ACPA+ (%)	OR (95% CI)	*P *value
						
*GSTM1-present*	73	*Ref*.	-	69	*Ref*.	-
*GSTM1-null*	79	1.45 (1.02 to 2.05)	0.039	69	1.00 (0.71 to 1.42)	0.981
						
Never smoking	69	*Ref*.	-	69	*Ref*.	-
Ever smoking	78	1.48 (0.97 to 2.26)	0.067	68	0.90 (0.62 to 1.30)	0.574
Former smoking	77	1.41 (0.91 to 2.19)	0.129	65	0.77 (0.52 to 1.14)	0.193
Current smoking	81	1.68 (0.98 to 2.88)	0.059	74	1.23 (0.76 to 1.99)	0.407
						
*SE-negative*	53	*Ref*.	-	54	*Ref*.	-
*SE-positive *(one or two alleles)	84	4.36 (2.98 to 6.37)	< 0.001	75	2.56 (1.77 to 3.70)	< 0.001
*SE-positive *(one allele)	79	3.23 (2.17 to 4.81)	< 0.001	68	1.77 (1.21 to 2.60)	0.003
*SE-positive *(two alleles)	93	10.65 (5.61 to 20.20)	< 0.001	92	10.27 (5.05 to 20.89)	< 0.001

^a^ACPA, anticitrullinated protein antibody; CI, confidence interval; *GSTM1*, *glutathione S-transferase Mu-1*; OR, odds ratio; *SE*, *shared epitope*; SONORA, Study of New-Onset Rheumatoid Arthritis; VARA, Veterans Affairs Rheumatoid Arthritis Registry. All analyses are age- and gender-adjusted. "*Ref.*" = referent group in each analysis.

**Table 3:** Associations of composite risk factors with ACPA positivity in patients with rheumatoid arthritis^a^

	VARA (*n *= 703)			SONORA (*n *= 610)		
	ACPA+ (%)	OR (95% CI)	*P *value	ACPA+ (%)	OR (95% CI)	*P *value
*GSTM1*/*SE*^a,b^						
Present/Negative	50	*Ref*.	-	59	*Ref*.	-
Null/Negative	56	1.26 (0.68 to 2.33)	0.456	48	0.63 (0.35 to 1.15)	0.135
Present/Positive	79	3.65 (2.09 to 6.40)	< 0.001	72	1.79 (1.05 to 3.03)	0.032
Null/Positive	88	7.30 (4.01 to 13.29)	< 0.001	77	2.29 (1.34 to 3.90)	0.002
	AP = 0.46 (0.20 to 0.73)			AP = 0.38 (0.00 to 0.76)		
	*P*_add _< 0.001			*P*_add _= 0.050		
	*P*_mult _= 0.246			*P*_mult _= 0.063		
*GSTM1*/Smoking^a^						
Present/Never	67	*Ref*.	-	72	*Ref*.	-
Present/Ever	75	1.42 (0.80 to 2.52)	0.227	67	0.72 (0.42 to 1.22)	0.223
Null/Never	72	1.35 (0.65 to 2.79)	0.421	67	0.75 (0.42 to 1.36)	0.349
Null/Ever	81	2.01 (1.13 to 3.55)	0.017	70	0.83 (0.49 to 1.42)	0.502
	AP = 0.12 (-1.41 to 1.65)			AP = 0.44 (-0.28 to 1.15)		
	*P*_add _= 0.881			*P*_add _= 0.231		
	*P*_mult _= 0.917			*P*_mult _= 0.245		
*SE*/Smoking^a^						
Negative/Never	55	*Ref*.	-	58	*Ref*.	-
Negative/Ever	53	0.90 (0.40 to 1.99)	0.788	51	0.73 (0.39 to 1.37)	0.329
Positive/Never	73	2.24 (0.97 to 5.16)	0.058	74	2.03 (1.08 to 3.82)	0.027
Positive/Ever	87	5.05 (2.31 to 11.05)	< 0.001	75	2.10 (1.17 to 3.78)	0.013
	AP = 0.58 (0.31 to 0.85)			AP = 0.16 (-0.34 to 0.66)		
	*P*_add _< 0.001			*P*_add _= 0.530		
	*P*_mult _= 0.054			*P*_mult _= 0.380		

^a^ACPA, anticitrullinated protein antibody; AP, attributable proportion; *GSTM1, glutathione S-transferase Mu-1*; *SE*, shared epitope; SONORA, Study of New-Onset Rheumatoid Arthritis; VARA, Veterans Affairs Rheumatoid Arthritis. All analyses are age- and gender-adjusted. ^b^Corresponding ORs and 95% for *GSTM1/SE *composite risk after further adjustment for ever smoking in VARA: Null/Negative OR = 1.22 (0.66 to 2.27); Present/Positive OR = 3.75 (2.13 to 6.59); Null/Positive OR = 7.34 (4.02 to 13.34); in SONORA, Null/Negative OR = 0.65 (0.36 to 1.18); Present/Positive OR = 1.80 (1.06 to 3.06); Null/Positive OR = 2.31 (1.36 to 3.95). "*Ref.*" = referent group in each analysis.

### *SE-GSTM1 *interactions

In the VARA registry, there was significant additive interaction between *SE *and *GSTM1 *status (AP, 0.46; 95% CI, 0.20 to 0.73; *P *< 0.001), an interaction that was evident, albeit of borderline significance, in SONORA (AP, 0.38; 95% CI, 0.00 to 0.76; *P *= 0.050). There was no evidence of a multiplicative *SE-GSTM1 *interaction in the VARA registry (*P *= 0.25), although the *P *value of the product term approached significance in SONORA (*P *= 0.06) (Table 3). These results were not changed for either cohort after further adjustments for cigarette smoking (Table 3).

**Figure 1:**
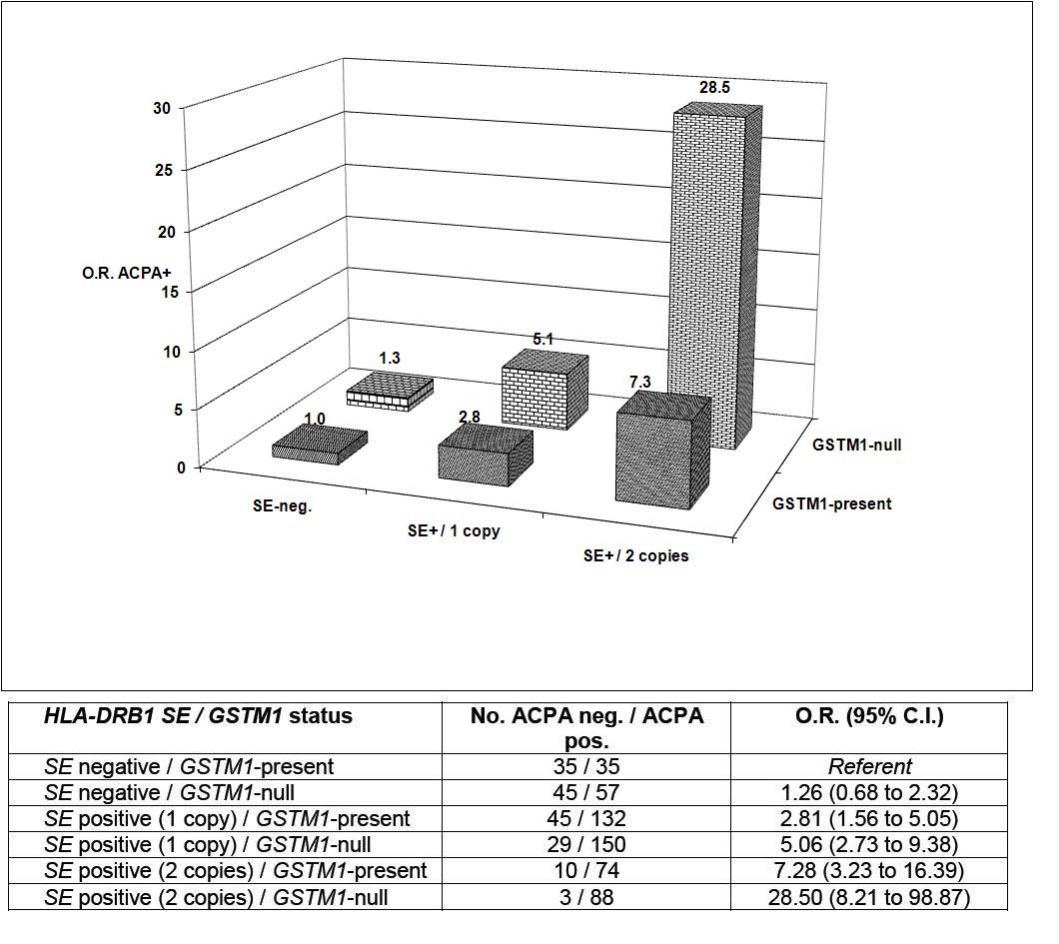
**Age- and gender-adjusted associations of composite *HLA-DRB1 SE *dose (0, 1 or 2 alleles) and *glutathione S-transferase Mu-1 *(*GSTM1*) status with anticitrullinated protein antibody (ACPA) positivity in Caucasian patients enrolled in the Veterans Affairs Rheumatoid Arthritis (VARA) registry (*n *= 703)**.

**Figure 2:**
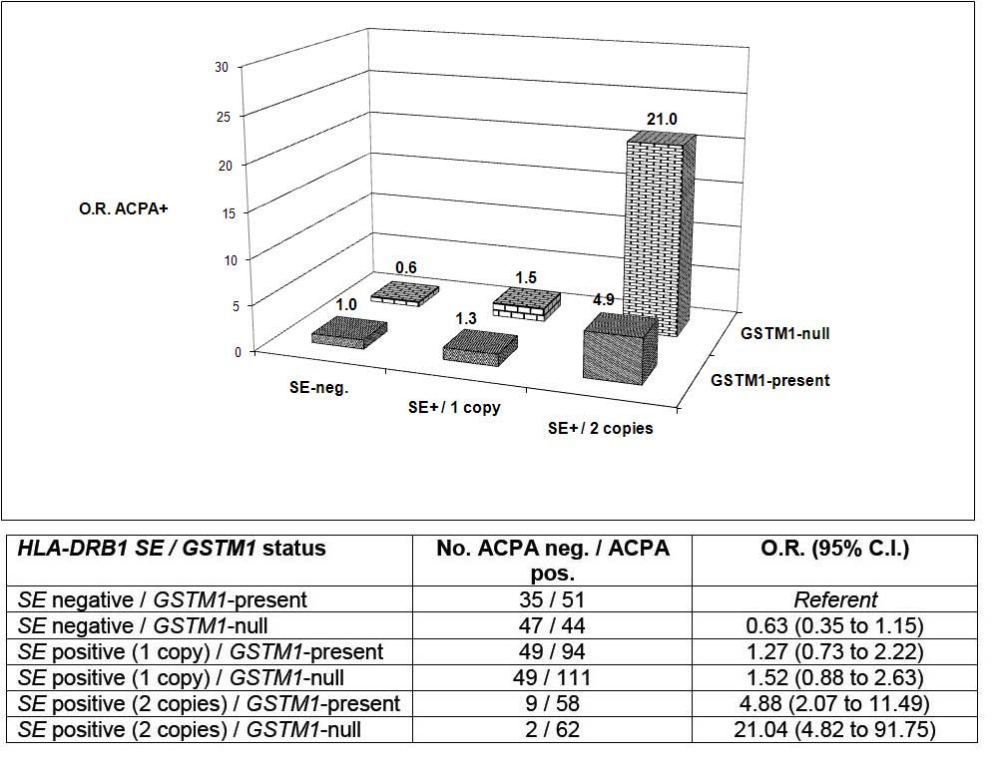
**Age- and gender-adjusted associations of composite *HLA-DRB1 SE *dose (0, 1 or 2 alleles) and *GSTM1 *status with ACPA positivity in Caucasian patients enrolled in SONORA (*n *= 610)**.

In exploratory analyses stratified by *SE *dose (0, 1 or 2 copies) rather than *SE *positivity, there were marked differences in the associations of composite risk factors of *GSTM1 *status and *SE *dose with ACPA positivity. Compared to individuals lacking both risk factors, *SE *homozygotes carrying the *GSTM1-null *genotype were ~28-fold more likely to be ACPA-positive in the VARA registry (OR, 28.50; 95% CI, 8.21 to 98.87) (Figure 1) and ~21-fold more likely to be ACPA-positive in SONORA (OR, 21.04; 95% CI, 4.82 to 91.75) (Figure 2).

### *GSTM1*-smoking and *SE*-smoking interactions

There were no significant additive or multiplicative interactions between *GSTM1 *status and smoking for ACPA positivity in either cohort (Table 3). In contrast, there was a significant additive interaction between *SE *positivity and ever smoking in the VARA registry, accounting for more than 50% of the overall risk of ACPA positivity in *SE*-positive smokers (AP, 0.58; 95% CI, 0.31 to 0.85; *P *< 0.001); there was also a nonsignificant trend to suggest multiplicative interaction (*P *= 0.054). Consistent with prior reports in SONORA [37], we observed no evidence of additive or multiplicative interactions between *SE *and ever smoking referent to ACPA positivity in this cohort. To explore the effect of smoking categorization on this finding, these analyses were repeated to examine for evidence of interaction between *SE *and current smoking (vs. never and former smoking combined). In these analyses, there was significant additive interaction between *SE *and current smoking (AP, 0.47; 95% CI, 0.13 to 0.82; *P *= 0.008), but no evidence of multiplicative interaction (*P *= 0.153) (data not shown).

## Discussion

Associations of *glutathione S-transferase *polymorphisms with RA have been the subject of several other investigations [12-15,38,39], although none of these have examined the association of *GSTM1 *status with ACPA-positive disease. This study is the first to show that the *GSTM1-null *genotype, present in approximately one half of all individuals of European ancestry, shows a significant biologic interaction with *HLA-DRB1 SE*-containing alleles with reference to the risk of ACPA positivity in RA. This is noteworthy, given the disease specificity (> 95%) of ACPA and the association of worse long-term outcomes in RA with ACPA seropositivity [22,23]. These results show that patients with both genetic risk factors (*HLA-DRB1 SE *and *GSTM1-null*) are two to seven times more likely to be ACPA-positive than patients lacking both risk factors. Furthermore, ~40% to 50% of the "excess" risk in this group is directly attributable to gene-gene interaction, an interaction that appears to be independent of smoking status. It is important to note that the magnitude of this interaction is similar to that previously reported to exist between *HLA-DRB1 SE *positivity and smoking [2,3]. The potential generalizability of these findings is further bolstered by its replication in two widely divergent RA cohorts: one composed primarily of men with long-standing disease and the other including primarily women with early-onset disease. These data are an important addition to studies showing significant additive interactions between *SE *and ever smoking in the risk of ACPA-positive RA [2,3]. Our results support the hypothesis that an oxidative environment promoted through the absence of functional *GSTM1 *enzyme potently enhances the risk of ACPA positivity in RA conferred by the presence of *HLA-DRB1 SE*.

Oxidative stress plays a pathogenic role in other autoimmune and inflammatory conditions, including systemic lupus erythematosus (SLE), scleroderma, diabetes and atherosclerosis [40]. Compared to those with functional *GSTM1*, individuals with the *GSTM1-null *genotype appear to be more prone to have increased levels of oxidative stress following exposure to select toxins [41]. Oxidation of nucleotides by reactive oxygen species increases the immunogenicity of DNA in SLE, generating autoantigens with significantly higher affinity for circulating autoantibodies [42]. In addition to modifying DNA and lipids, oxidative stress promotes the formation of neoantigens through posttranslational peptide modification. Bang *et al*. [43] have shown that oxidation of citrullinated vimentin, implicated as an autoantigen in RA, leads to substantially increased antibody reactivity to this antigen in RA.

Our results complement the prior findings of Klareskog *et al*. [2], who reported that patients who had ever smoked and were homozygous for *SE *were 21 times more likely to develop ACPA-positive RA compared to *SE-negative *patients who had never smoked. Results from the Swedish case control study [2] differed from an analysis of three North American cohorts including SONORA [37], which found no evidence of interaction between *SE *and ever smoking in SONORA and only weak evidence of interaction in one of the two other cohorts examined. In these two other cohorts, but not in SONORA, ever smoking showed a borderline association with ACPA positivity with ORs approaching 1.4 [37]. Although it was not statistically significant, we found a similar association of ever smoking with ACPA positivity in the VARA registry with an OR of 1.48, suggesting that our study was underpowered to detect this association because of the relatively small proportion of never smokers in the VARA Registry.

Differences in these reports (and differences between the VARA registry and SONORA) could relate to population heterogeneity, including differences in gender distribution and cumulative smoking exposure. Compared to women, men have been shown to have a higher penetrance of *HLA-DRB1 *[44], are more likely to smoke and (among smokers) are more likely to be categorized as heavy smokers [45]. Differences in smoking exposure may be salient here, given findings from a separate North American study showing that *SE*-smoking interactions in the risk of seropositive RA (a combined rheumatoid factor (RF)/ACPA-positive phenotype) were limited to individuals with heavy smoking (> 10 pack-years) [46]. The importance of quantifying cumulative smoking exposure has also recently been shown among African Americans with RA risk limited to those with more than 10 pack-years of exposure [47]. Cumulative smoking exposure was not available in the present study involving the VARA registry and SONORA, precluding such analyses. Underscoring the potential importance of accounting for cumulative exposure, we observed significant *SE*-smoking interactions in SONORA when smoking exposure was dichotomized as current vs. noncurrent rather than ever vs. never, with the "current" category likely to account for individuals with greater lifelong smoking exposure.

These results differ from a prior study showing significant multiplicative interactions between *GSTM1-null *status and smoking in RA disease risk [12], an effort that did not include examinations of *GSTM1-SE *interactions. In the present study, we found no evidence of significant interaction (multiplicative or additive) between *GSTM1-null *and smoking in ACPA positivity, a phenotype that was not examined in the prior nested case control analysis from the Iowa Women's Health Study [12]. It is possible that in the present study we simply lacked sufficient power to detect this interaction. Differences in study design (case only vs. case control) and study populations (smoking prevalence and predominantly male vs. female patients) may also help explain these discrepant study results.

Controversy and uncertainty remain regarding the most appropriate manner in which to model gene-gene and gene-environment interactions [48]. In contrast to prior studies that have examined smoking-*SE *interactions in RA risk by calculating only measures of additive interaction [2,49], we have examined measures of both additive and multiplicative interaction. Multiplicative interaction refers to the inclusion of a product term in regression analyses to generate an optimal fit of the data in the statistical model. It is important to note that the absence of multiplicative interaction does not exclude the existence of important biologic interactions. For example, the present study shows that at least one pathway to ACPA positivity in RA requires the presence of two risk factors (that is, *GSTM1-null *and *HLA-DRB1 SE*).

Although they involved two large independent cohorts, our analyses were limited to two-way interactions. We lacked the sample sizes even after combining cohorts that would be necessary to examine more complex interactions, including analyses of *GSTM1-SE *stratified by smoking status. Future analyses of this sort with larger patient populations will be essential not only in replicating our findings but also in providing critical insight into mechanisms underpinning these observed interactions. Although this study included a case-only approach, ACPA positivity is increasingly recognized as a distinct disease phenotype in RA. Indeed, the well-defined associations of cigarette smoking and *HLA-DRB1 SE *with RA in European populations apply only to ACPA-positive disease and do not apply to seronegative disease [2]. Because of the limited sample sizes in subgroups of interest, our study did not include analyses of interactions of distinct *HLA-DRB1 *subtypes with *GSTM1*. Recent findings have shown that different *01 and *04 subtypes appear to contribute equally to *SE*-smoking interactions in ACPA-positive RA [50], suggesting that analyses of specific *SE *subtypes may yield limited incremental information.

## Conclusions

The *GSTM1-null genotype*, observed in approximately 50% of individuals of European ancestry, shows significant interactions with *HLA-DRB1 SE *alleles in ACPA positivity among patients with RA. Future studies will be needed to explore precisely how *GSTM1 *and other antioxidant enzymes influence disease expression in RA. Along with other recent reports, this work emphasizes the need for the simultaneous investigation of multiple genetic and environmental factors to better understand the pathogenic contributions of these elements to the development and progression of RA with potential application to other autoimmune diseases.

## Abbreviations

ACPA: anticitrullinated protein antibody; ACR: American College of Rheumatology; AP: attributable proportion; CI: confidence interval; CYP1a1: cytochrome p450 1a1; GSTM1: *glutathione S-transferase Mu-1*; HLA: human leukocyte antigen; OR: odds ratio; PCR: polymerase chain reaction; RA: rheumatoid arthritis; SE: *shared epitope*; SLE: systemic lupus erythematosus; SONORA: Study of New-Onset Rheumatoid Arthritis; SSOP: specific oligonucleotide probes; VA: Veterans Affairs; VARA: Veterans Affairs Rheumatoid Arthritis.

## Competing interests

The authors declare that they have no competing interests.

## Authors' contributions

TRM was involved in all aspects of study conception, design, analysis, interpretation and report generation and provided final approval of the version of the submitted manuscript. TRM had full access to all of the study data and had final responsibility for the decision to submit the manuscript. SLB, LH, PKG, JAN, FY, KAG, TDLV, GMT, KDM, JRO'D, AMR, RH, LC, DSJ, GK, JSR, GWC and KKB were involved in data acquisition, analysis and report drafting and provided final approval of the submitted manuscript. LAC was involved in data interpretation and report generation and also provided final approval of the submitted manuscript draft.
